# Comparison of Up‐on‐the‐Toes Stand Test Performance in Young Adults and Older Adults With Diabetic Peripheral Neuropathy

**DOI:** 10.1002/jfa2.70112

**Published:** 2025-12-17

**Authors:** John G. Buckley, Asma Sabouni, Andre L. F. Rodacki, Steve Brown, Neil D. Reeves

**Affiliations:** ^1^ School of Engineering, Faculty of Engineering and Digital Technologies University of Bradford Bradford UK; ^2^ Department of Physical Education Federal University of Paraná Curitiba Paraná Brazil; ^3^ Department of Sport and Exercise Science, Faculty of Science and Engineering Manchester Metropolitan University Manchester UK; ^4^ Medical School, Faculty of Health and Medicine Lancaster University Lancaster UK

**Keywords:** ankle function, balance control, postural stability, up‐on‐the‐toes

## Abstract

**Background and Purpose:**

Proficient ankle functioning provides a key contribution to everyday activities, such as walking and stair ascent and descent, where many falls occur. The up‐on‐the‐toes stand test (UTTS), involves rising from standing to an up‐on‐the‐toes position and holding it for 5 s, before lowering back to standing. Here, we explore whether the balance‐related UTTS test scores differ between two groups with expected differences in ankle functioning, that is, between older adults with diabetic peripheral neuropathy (DPN) in comparison to young healthy adults.

**Design:**

Case–control study.

**Methods:**

On a force platform, 13 older adults with DPN and 14 young adults completed repeated UTTS. Outcome measures were the peak forwards and backwards centre of pressure (CoP) velocity when rising and lowering, the average CoP displacement and variability in CoP velocity when holding the up‐on‐the‐toes position, and the time it was held.

**Results:**

In older adults with DPN compared to young adults, the forwards CoP velocity and displacement when rising up‐on‐the‐toes were reduced (*p* < 0.001), indicating a slower speed and range of movement; variability in CoP velocity when up‐on‐the‐toes was greater, indicating reduced stability (*p* = 0.021); and time up‐on‐the‐toes was shorter (*p* = 0.002), indicating a reduced ability to hold this position.

**Conclusions:**

Findings indicate that older adults with DPN had poorer UTTS performance in comparison to young adults. This suggests the test might be useful for highlighting how deficits in ankle mobility and function can impact balance when moving up‐on‐the‐toes. Future research should compare (a) older DPN patients with healthy older adults and (b) young adults with older adults, to determine whether UTTS test can validly assess age‐related decline. In addition, case–control designs within the same age group are necessary to determine whether UTTS test can distinguish disease‐specific balance deficits.

## 1 Introduction

Increased standing postural sway is associated with an increased risk of falling in older adults [1]. Hence, postural control assessment is routinely used to identify elderly individuals with an increased risk of falling [2, 3, 4, 5]. Such assessment typically involves evaluating postural stability/sway during quiet standing [6, 7] or postural control during sit‐to‐stand transitions [8, 9, 10, 11] or during reaching [12, 13, 14, 15]. In these tasks, the base of support is the full plantar surface of the two feet on the ground. However, these tasks pose minimal postural balance demands, particularly compared to many locomotor activities of daily living. Most daily activities involve phases in which the base of support is reduced to one foot, such as the single‐support phase of walking. Additionally, specific sub‐phases further constrain the base of support to the forefoot or metatarsal region, such as during the terminal portion of single‐limb support in stair descent. During this sub‐phase, the body centre of mass (CoM) is being lowered whereas the contralateral leading limb is extending forward and downward to initiate contact with the step below, which places great demands on balance control [16]. Although balance control depends on several aspects [17, 18], most actions required to rise and lower the CoM rely greatly on the functioning and strength of ankle muscles [19, 20]. Therefore, assessing balance control during a relatively controlled task requiring substantial contribution of the foot and ankle under an enforced reduction in the base of support may be useful for identifying balance impairments.

Functional ankle output (or proficient ankle functioning) refers to the ability of the foot–ankle complex to perform the movements needed for everyday activities [21]. It is widely recognised that individuals with diabetes have compromised foot–ankle functioning due to factors associated with peripheral vascular disease (also referred to as diabetic peripheral neuropathy, DPN), including reduced foot–ankle flexibility [22], reduced ankle/plantarflexion strength [23, 24, 25, 26, 27, 28], low physical functioning and/or everyday mobility [29, 30, 31] and poorer sensory‐motor control and balance [24, 32]. These factors may help explain the high incidence of falls in older adults with DPN compared to age‐matched counterparts [32, 33, 34, 35]. It also underscores that evaluation of dynamic balance ability during a simple yet challenging movement task that relies on substantial contribution of the foot and ankle could provide a simple assessment tool for determining falls risk in older adults with DPN and other patient groups who have compromised ankle functioning.

**FIGURE 1:**
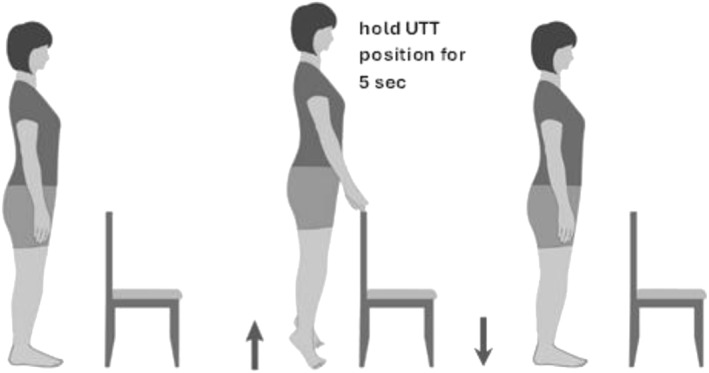
Depiction of the UTT‐stand test. Participants stood with feet shoulder width apart, on a force platform (not shown) with their gaze directed forwards. After 4–5 s of ‘quiet’ standing to establish a baseline GRF, participants rose to an up‐on‐the‐toes position and attempted to hold the position for 5 s, before returning to a ‘feet flat’ standing position. For those with balance issues, a standard office‐type chair can be placed in front, allowing participants to place index fingers lightly on the back of the chair to avoid losing balance. The chair is only used as a precaution when/if necessary and not as an assistive aid to provide bodyweight support. Note, in the present study, none of the participants requested use of a chair.

The up‐on‐the‐toes stand test (UTTS [36] and Figure 1), also known as the rise to toes test [37], is a relatively controlled yet challenging movement task. Performing a UTTS relies on the strength and function of the foot and ankle and thus may be useful for identifying how deficits in foot–ankle functioning affect balance. Previous research has shown that the relative timing of postural adjustments and voluntary movement when performing a UTTS is impaired in Parkinsons patients in comparison to healthy young adults, with such impairments diminishing when Parkinsonian patients are on levodopa [37]. Other research, by the same group, found that inducing fear of falling in young healthy participants impaired their UTTS performance, mainly reflected by alterations in both the timing and magnitude of the anticipatory postural adjustments [38]. More recent work has assessed the importance of vision in performing a UTTS and showed that under occluded vision conditions, the rise‐to‐toes speed is slower, whereas the stability when holding the UTTS position is diminished and the speed of movement when returning to standing is increased (indicating less control) [39, 40]. These previous studies have been mostly concerned with understanding how centrally mediated control and/or sensory impairment impact movement control. It is important to highlight that the UTTS differs from standard static tests, as it provides a more functional assessment of dynamic balance because it requires active plantarflexion and ongoing control of whole‐body momentum. The test may better mirror everyday mobility tasks such as stair negotiation demands, which cannot be fully captured by static tests (which are primarily designed as an index of quiet‐stance stability).

The focus of the current study was to explore whether the balance‐related UTTS test scores differ between two groups with expected differences in ankle functioning, that is, between older adults with DPN in comparison to healthy adults (young adults). A secondary aim was to determine how the speed of performing the UTTS affected test outcomes in older adults with DPN compared to young adults. We hypothesised that due to assumed weaker musculature and/or poorer neuromuscular control predominantly at the ankle, older adults with DPN would have a slower speed of movement when rising UTT, poorer stability when holding the UTT position and faster/uncontrolled speed of movement when lowering back to the feet flat position. The study also aimed to highlight the extent to which UTTS test parameters (e.g., CoP velocity and displacement when rising to the toes and CoP velocity variability when up‐on‐the‐toes) are associated with ankle functioning parameters (e.g., angular displacement and velocity during plantar and dorsiflexion) during the UTTS test.

## 2 Materials and Methods

### 2.1 Design

Type of study: A case–control study examining whether the UTTS test can identify balance performance differences in older adults with DPN compared to young healthy adults.

Recruitment approach: Convenience sampling based on who (meeting the inclusion criteria) from a local Diabetes Centre and the local community volunteered to take part within a limited period. This resulted in the recruitment of 13 older adults with DPN and 14 young adults. Statistical power estimate: A post hoc power calculation was performed ([41]; GPower 3.1 software) using the group means in peak forwards CoP velocity when moving up‐on‐the‐toes. The alpha error probability was set at 0.05, and the correlation between measures was considered as 0.75, with a nonsphericity correction of 1. This retrospective calculation indicated the study's power was 89% (which is higher than the 80% i.e. generally considered a good and acceptable level [41]). Study location: University‐based research laboratories. Independent variables: The group each participant was recruited into, based on whether they were older adults diagnosed with diabetic peripheral neuropathy or young healthy adults (with no diabetic peripheral neuropathy). Dependent variables: Peak forwards CoP velocity when moving up‐on‐the‐toes; average forwards displacement of CoP when holding the up‐on‐the‐toes position relative to position during neutral standing; the variability in AP‐CoP velocity when holding the up‐on‐the‐toes position; the CoP peak backwards velocity when returning to standing; the time of holding the up‐on‐the‐toes position. How these variables were defined/determined are outline in section ‘2.5.1 CoP Analysis’ below.

### 2.2 Participants

Thirteen individuals with diabetic peripheral neuropathy (older adults with DPN; age 70.8 ± 10.1 year; height 1.78 ± 0.04 m; and mass 91.4 ± 16.3 kg) and 14 healthy adults (young adults; age 43.8 ± 22.5 year; height 1.75 ± 0.08 m; mass 84.7 ± 11.3 kg) gave written informed consent to take part. The older adults with DPN group had an average (± SD) duration since diagnosis of 15 (5) years and a Vibration Perception Threshold of 27 (11) V. *T*‐tests indicated no differences between groups in body mass (*p* = 0.09) or stature (*p* = 0.11). The older adults with DPN group were older than the control group (*p* < 0.001). Ethical approval was granted by the institutional bioethics committee (approval number E.119) and the NHS Research Ethics Committee (18/NW/0274), and the tenets of the Declaration of Helsinki were observed. Participants were asked to wear flat‐soled, comfortable shoes and their corrective spectacles or contact lenses (if usually worn for walking).

Older adults with DPN were chosen because they are known to have impaired foot and ankle functioning compared to young adults (see examples outlined in the Introduction). Although this led to an age difference between groups, this was in keeping with achieving the study's main purpose, as it further ensured there would be differences in UTTS test performance between groups.

### 2.3 Protocol

Participants stood with feet side‐by‐side, approximately shoulder width apart, on a force platform (AMTI OR6‐7: Boston, MA, USA or Kistler 9281E, Winterthur, Switzerland) with their gaze directed forwards. After approximately 4–5 s of ‘quiet’ standing to establish a stable baseline ground reaction force (GRF), participants were asked to rise to an up‐on‐the‐toes position and to try and hold the position for 5 s, before returning to a ‘feet flat’ standing position (Figure 1). A researcher counted out loud (up to 5, slowly and deliberately) so that participants could track how long they held the up‐on‐the‐toes position. Although counting out loud encouraged participants to maintain the up‐on‐the‐toes position for 5 s, not all participants for all trials were able to hold the position for the full 5 s. Trials were repeated three times. This condition was termed ‘FREE’, since participants performed using their preferred self‐selected rising speed. In a separate trial of the task, participants were asked to repeat the task one further time but were required to rise on to their toes as quickly as possible, which was termed ‘FAST’. Because balance control is more challenging when rising up‐on‐the‐toes quickly, participants were not given a specific time to hold the up‐on‐the‐toes position and were allowed to return to a ‘feet flat’ standing position in their own time.

### 2.4 Data Acquisition and Processing

Ground reaction force (GRF) data and lower limb kinematic data (using a multi‐camera motion capture system, Vicon Mx, Oxford Metrics Ltd., Oxford, UK) were collected at 200 Hz. Reflective markers (14 mm diameter) were attached bilaterally either directly onto clothing or shoes, as per the lower body Helen‐Hayes marker set [42]. Marker trajectory data were labelled and gap‐filled within the Nexus software (Oxford Metrics). Gap filling was completed either using ‘pattern fill’ of the trajectory from another marker attached to the same segment as the missing marker or using the spline fill option if ‘pattern fill’ was not possible. Using the Plug‐In‐Gait software within Nexus (Oxford Metrics Ltd), a 3D link‐segment lower body model incorporating anthropometric data was embedded for each participant. The marker trajectory and joint angular data were low‐pass filtered (8 Hz cut‐off). The GRF data were also low‐pass filtered (20 Hz cut‐off). CoP coordinate data, along with ankle angular displacement data, were subsequently exported in ASCII format for further analysis. All outcome measures were determined for each trial, using ‘in‐house’ analysis routines written in Visual Basic.

### 2.5 Data Analysis

#### 2.5.1 CoP Analysis

**FIGURE 2:**
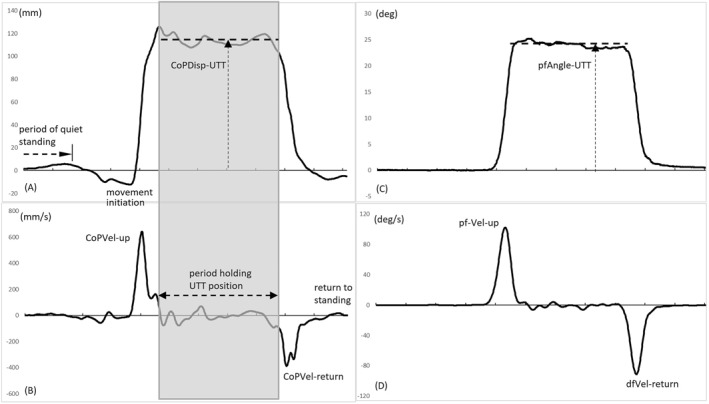
Ground reaction force data (left‐hand panels) and ankle kinematics (right‐hand panels) from a sampler trial, showing measurement of the key parameters for the UTTS test. (A) CoP displacement trajectory, (B) CoP velocity trajectory, (C) the ankle angular plantarflexion displacement trajectory and (D) the ankle angular plantarflexion velocity trajectory. NB, the CoP displacement and ankle angular displacement trajectories have been normalised by subtracting from them, respectively, the average CoP displacement value and average ankle angular displacement value determined for the period of quiet standing prior to movement initiation. See text for definition of each labelled variable.

The following variables were analysed from the GRF data (Figure 2A,B):

— Peak forwards CoP velocity when moving up‐on‐the‐toes (CoPVel‐up). Determined as the peak AP‐CoP velocity in the anterior direction following movement initiation.
— Average forwards displacement, from neutral standing position, of the CoP when holding the up‐on‐the‐toes position (CoPDisp‐UTT). Determined by subtracting the average AP‐CoP position when standing stationary, from the average AP‐CoP position when holding the up‐on‐the‐toes position. Holding the up‐on‐the‐toes position was deemed to have started when the CoP forwards velocity slowed to less than 150 mm/s after its peak during rising. The end of holding the up‐on‐the‐toes position was determined as the instance the CoP velocity increased in the backwards direction to greater than −150 mm/s following the start of holding the up‐on‐the‐toes position.
— The variability in AP‐CoP velocity when holding the up‐on‐the‐toes position (SD‐CoPVel‐UTT). Determined as the standard deviation in AP‐CoP velocity for the period of holding the up‐on‐the‐toes position.
— The CoP peak backwards velocity when returning to standing (CoPVel‐return). Determined as the peak AP‐CoP velocity in the posterior direction when returning to standing.
— The time of holding the up‐on‐the‐toes position (time‐UTT). The time from the start to the end of holding the up‐on‐the‐toes position.

#### 2.5.2 Ankle Kinematics

The following variables were measured to describe the ankle kinematics (Figure 2C,D):

— The peak plantarflexion angular velocity when moving up‐on‐the‐toes (pfVel‐up). Determined as the peak ankle angular velocity in positive (plantarflexion) direction, following movement initiation.
— The peak dorsiflexion angular velocity when returning to standing (dfVel‐return). Determined as the peak ankle angular velocity in negative (dorsiflexion) direction, when returning to standing with feet flat.
— The average displacement in plantarflexion for when up‐on‐the‐toes (pfAngle‐UTT). Determined by subtracting the average plantarflexion angle when standing stationary, from the average plantarflexion angle when holding the up on the toes position.
— The variability in ankle angular velocity when holding the up‐on‐the‐toes position (SD‐pfVel‐UTT). Determined as the standard deviation in ankle angular velocity for the period of holding the up‐on‐the‐toes position.

We initially considered measuring the height at which the heels were raised when obtaining the UTT position but subsequently reasoned that the height raised would depend on the length (size) of a person's foot. Hence, instead of assessing the height the heels were raised, we decided to assess the angle the feet attained when reaching the UTT position (determined as plantarflexion angle).

### 2.6 Statistical Analysis

Given that the extent to which the feet were plantarflexed in obtaining the UTT position was self‐selected, it is possible that any group outcome differences found might be influenced by group differences in the consistency in performing the repeated UTTS trials. Therefore, the variability in the plantarflexion angle across the customary speed trials was determined for each participant and then averaged across each group (Note, inter‐trial variability is only relevant to the customary speed trials because the FAST speed trial was only performed once). This analysis yielded group average variability values of 9.0° and 10.1° for older adults with DPN and young adults, respectively, and a paired *t*‐test indicated that there was no significant difference in plantarflexion angle variability between groups (*p* = 0.20). This highlights the consistency in performing the repeated UTTS trials was similar between groups.

The UTTS outcome measures were compared between groups using mixed‐mode repeated measures ANOVA with speed as a within‐subjects factor and group as a between‐subjects factor. Analyses were undertaken using the JASP software (Version 0.18.1), and the significance level was set at *p* < 0.05. Outcome values for the FREE speed were the average values recorded across the 3 FREE speed repetitions. Data normality was confirmed by Shapiro–Wilk tests.

As analysis of the UTTS test is based on outcomes from the force platform, no statistical comparisons of limb kinematics between the groups were undertaken. Limb kinematics during the UTTS were determined to highlight the degree to which UTTS test outcomes (i.e., CoP measurement parameters) are associated with ankle functionality. To this end, scatterplots of the UTTS and ankle kinematic measures, using data for all participants, were created to highlight the association between the UTTS test outcomes and ankle functional behaviour.

To further determine the extent to which UTTS test outcomes are associated with ankle functionality, Spearman correlation coefficients were calculated between key UTTS measures and the related ankle kinematic measure. R‐values of < 0.35 represent low to weak correlation; those between 0.36 and 0.67 represent moderate correlation and those between 0.68 and 1 represent a strong correlation [43].

## 3 Results

Table 1 shows group mean UTTS measures for the FREE and FAST conditions.

**TABLE 1:** Group mean (± SD) UTTS test metrics when performing the test (a) at freely chosen speed and (b) at fast speed.

(a) Free speed (average) (b) Fast speed	Young adults		Older adults with DPN		ANOVA
	Mean	SD	Mean	SD	
CoPVel‐up (mm/s)					**Grp = 0.001** Speed < 0.001 Int = 0.59
(a)	524.8	148.9	310.8	197.4	
(b)	967.9	241.4	698.3	265.3	
CoPVel‐return (mm/s)					Grp = 0.27 Speed = 0.013 Int = 0.86
(a)	428.4	157.0	367.9	281.2	
(b)	575.7	211.7	496.6	162.5	
CoPDisp‐UTT (mm)					**Grp** **<** **0.001** Speed = 0.022 Int = 0.60
(a)	93.9	17.8	64.7	30.0	
(b)	110.4	29.1	75.2	20.1	
SD‐CoPVel‐UTT (mm)					**Grp = 0.021** Speed = 0.65 Int = 0.32
(a)	48.4	14.1	76.5	31.8	
(b)	60.3	34.4	72.0	34.0	
Time‐UTT (s)					**Grp = 0.002** Speed < 0.001 Int = 0.26
(a)	4.2	1.2	2.7	0.8	
(b)	3.0	1.6	1.9	0.7	

*Note: CoPVel‐up*, the peak forwards CoP velocity when moving up‐on‐the‐toes. *CoPVel‐return*, the CoP peak backwards velocity when returning to standing. *CoPDisp‐UTT*, average forwards displacement of CoP when holding the up‐on‐the‐toes position. *SD‐CoPVel‐UTT*, the variability in AP‐CoP velocity when holding the up‐on‐the‐toes position. *time‐UTT*, the time of holding the up‐on‐the‐toes position.

### 3.1 Assessment of CoP Parameters

The interactions between the main effects of group and condition were nonsignificant for all the UTTS outcomes (*p* ≥ 0.26). The group (older adults with DPN vs. young adults) and condition (FREE vs. FAST) effects are highlighted below.

#### 3.1.1 Group Effects in CoP Parameters

When rising up‐on‐the‐toes, the peak forwards CoP velocity and CoP displacement were reduced for older adults with DPN compared to young adults in both speed conditions (*p* < 0.001). Across both speeds, the variability in CoP velocity when holding the up‐on‐the‐toes position was greater, indicating reduced stability, for older adults with DPN compared to young adults (*p* = 0.021). Across both speed conditions, the time the up‐on‐the‐toes position was held was shorter for older adults with DPN compared to young adults (*p* = 0.002). The peak backwards CoP velocity when returning to standing was not significantly different between groups (*p* = 0.27).

#### 3.1.2 Speed Effects in CoP Parameters

The peak forwards CoP velocity when rising up‐on‐the‐toes was greater in both groups for the FAST compared to FREE speed trials (*p* < 0.001), and the forwards CoP displacement was also greater for the FAST compared to FREE speed trials (*p* = 0.022). For both groups, the variability in CoP velocity whilst holding the up‐on‐the‐toes position was unaffected by the test speed condition (*p* = 0.65). Across both groups, the time the up‐on‐the‐toes position was held was shorter for the FAST compared to FREE trials (*p* < 0.001), and the peak backwards CoP velocity when returning to standing was greater for FAST compared to FREE trials (*p* = 0.013).

### 3.2 Association of UTTS Parameters With Limb Kinematics

**FIGURE 3:**
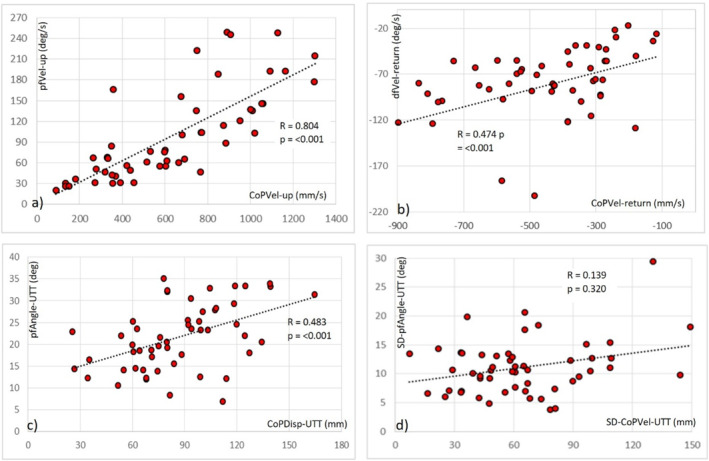
Scatter plots of UTTS test measures plotted against the related ankle kinematic measures: (a) peak forwards CoP velocity (CoPVel‐up) against peak plantarflexion angular velocity (pfVel‐up); (b) peak backwards CoP velocity (CoPVel‐return) against peak dorsiflexion angular velocity (dfVel‐return); (c) CoP forwards displacement (CoPDisp‐UTT) against plantarflexion angular displacement (pfAngle‐UTT) and (d) variability in CoP velocity (SD‐CoPVel‐UTT) against variability in plantarflexion angular velocity (SD‐pfVel‐UTT). The *R*‐values for the inserted linear regression lines indicate the correlation between the UTTS test measure and the ankle kinematic measure.

Figure 3 shows the scatterplots of each UTTS measure plotted against the related ankle kinematic measures. For the period of rising‐on‐to‐the‐toes, there was a strong correlation between the peak forwards CoP velocity and peak plantarflexion angular velocity (*R* = 0.80, *p* < 0.001 and Figure 3a). There was a moderate correlation between peak backward CoP velocity and peak dorsiflexion angular velocity, when returning to standing (*R* = 0.47, *p* < 0.001 and Figure 3b). There was also a moderate correlation between the forwards CoP displacement and the plantarflexion angular displacement (*R* = 0.48, *p* < 0.001 and Figure 3c) and weak correlation between the variability in CoP velocity and variability in plantarflexion angular velocity (*R* = 0.14, *p* = 0.32 and Figure 3d), when holding the up‐on‐the‐toes position.

## 4 Discussion

The aim of this study was to explore whether balance‐related UTTS test scores differ between groups with expected differences in ankle functioning, that is, between older adults with DPN in comparison to young adults. A secondary aim was to determine how the speed of performing the UTTS affected test outcomes in older adults with DPN in comparison to young adults. We hypothesised that due to assumed weaker musculature and/or poorer neuromuscular control predominantly at the ankle, older adults with DPN would have a slower speed of movement when rising UTT, poorer stability when holding the UTT position and faster/uncontrolled speed of movement when lowering back to the feet flat position.

Findings indicate that older adults with DPN had poorer UTTS performance in comparison to young adults; with a reduced forwards CoP velocity and displacement when rising up‐on‐the‐toes (indicating a slower speed and range of movement), greater variability in CoP velocity when holding the UTT position (indicating reduced stability) and a decreased time holding the UTT position. These findings support our hypotheses, and they demonstrate that the UTTS test can readily differentiate between groups with expected differences in ankle functioning. However, it is important to acknowledge that the group differences in UTTS test performance found in the present study may not be directly reflective of differences in foot–ankle functioning because we did not directly assess the strength and function of foot and ankle, but instead assumed older adults with DPN would have impaired strength and function as previous research has indicated [22, 23, 24, 25, 26, 27, 28, 29, 30, 31]. This means caution is needed in generalising the results to all those suffering with DPN and/or to all those who have impaired foot–ankle functioning.

When rising up‐on‐the‐toes, the AP‐CoP location will move forwards from its average location during standing (1–2 cm anterior of the ankles [44, 45]) to be aligned with or forward of the base of the toes (i.e., just anterior of the metatarsophalangeal joint). Along with a reduced CoP velocity when rising up‐on‐the‐toes (on average by 214 and 270 mm/s for FREE and FAST conditions, respectively), older adults with DPN also had a reduced CoP average displacement compared to young adults when holding the up‐on‐the‐toes position in both speed conditions (on average by 29.2 and 35.1 mm for FREE and FAST conditions, respectively; Table 1). These findings highlight that older adults with DPN were slower than the young adults rising on to their toes and did not move as far forward on to their toes as the young adults. This is likely due to older adults with DPN’ reduced flexibility and ankle strength [22, 23, 24, 25, 46]. This corroborates previous research showing that a reduction in hallux plantarflexion strength leads to poorer balance [47]. It is also plausible that the older adults with DPN had poorer sensory‐motor control and balance compared to the young adults [24, 32], which made it more difficult for them to achieve the same metacarpophalangeal dorsiflexion compared to the young adults. The reduced CoP displacement (and hence reduced CoM movement) in older adults with DPN when moving up on to their toes may also relate to a desire to constrain movement of the CoM due to a reduced ability to maintain control of the CoM during movements performed over a large range. The slower speed of movement and accompanying reduced forwards movement when rising onto the toes, supports our hypothesis that due to having weaker ankle musculature and/or poorer neuromuscular control, older adults with DPN would have a slower speed of movement when rising UTT.

We also hypothesised that older adults with DPN would have poorer stability when holding the UTT position and faster/uncontrolled movement speed when lowering back to standing. Older adults with DPN did indeed have significantly poorer stability (i.e., increased instability) when holding the UTT position (i.e., had increased variability in AP‐CoP velocity), and consequently, they could not sustain the up‐on‐the‐toes position for as long as the young adults did. However, although older adults with DPN returned to standing more quickly (i.e., had a higher CoPVel‐return) compared to young adults, at both FREE and FAST speeds, differences between groups were nonsignificant. The increased instability (and hence greater balance impairment [48]) in older adults with DPN when holding the up‐on‐the‐toes position supports our hypothesis and is likely due, at least in part, to them having weaker ankle musculature and/or poorer sensory‐motor neuromuscular control [24, 47]. The reduced time older adults with DPN spent holding the UTT position (time‐UTT) in comparison to young adults, for both the FREE and FAST speed conditions (by 1.5 and 1.1 s for FREE and FAST speeds, respectively), highlights further that the older adults with DPN had difficulty holding the UTT position on a reduced base of support (note, participants were asked to try to hold the UTT position for 5 s). The difficulty older adults with DPN had in maintaining the UTT position for 5 s was likely related to the increased instability (SD‐CoPVel‐UTT) when holding the UTT position, which caused them to relinquish the UTT position sooner.

As indicated, there was no significant group difference in the speed of movement when lowering back to standing. Given the significant group difference in CoP displacement when rising on‐to‐the‐toes (which indicated older adults with DPN did not move as far forwards onto the toes as young adults did), the lack of group differences in return speed suggest young adults must have exerted more control than older adults with DPN did because they returned to flat‐foot standing from a more forward position (i.e., they moved over a greater distance) but this did not lead to an increase in lowering speed. The reduced control exerted by the older adults with DPN when returning to standing is further highlighted by looking at the velocity of lowering relative to rising. If the lowering speed is expressed as a percentage of the rising speed, that is, ((CoPVel‐return/CoPVel‐up)*100), it signifies that older adults with DPN had a significantly greater relative lowering speed than young adults at both FREE (148% older adults with DPN, vs. 83% young adults) and FAST (81% older adults with DPN, vs. 62% young adults) speeds (*P* < 0.05). Having a greater relative lowering speed suggests that the control (muscle action) exerted during the eccentric lowering phase (return to standing) was reduced compared to the control (muscle action) exerted during the concentric rising phase (rise to UTT position). Why older adults with DPN exerted less relative control during lowering compared to that exerted by young adults may be indicative of them becoming more fatigued during the UTTS hold period (due to their increased instability during this period) resulting in them have poorer relative control during lowering compared to young adults.

To highlight the extent to which UTTS test scores are associated with ankle functioning, the current study determined the relationship (i.e., correlation) between UTTS test outcomes and ankle kinematics. The study found that CoP measures (i.e., UTTS outcomes) varied from being moderately to strongly significantly correlated with the ankle kinematic measures (correlation range from *r* = 0.47–0.80). This highlights that UTTS outcomes are reflective of the control exerted by the foot–ankle during this movement control task. The only UTTS outcome measure that was not significantly correlated to ankle kinematics was the variability in AP‐CoP velocity when holding the UTT position. This outcome parameter, which reflects stability when up‐on‐the‐toes, was found to have no association with the variability in ankle angular velocity when holding the UTT position. The lack of a correlation between these two measures may be related to the variability in AP‐CoP velocity being reflective of postural control of the whole‐body CoM, whereas the variability in ankle angular velocity is reflective of more localised control at the ankle.

Much previous work has highlighted that evaluation of the postural stability/sway during quiet ‘flat‐foot’ standing can be used to identify elderly individuals who have an increased falling risk [1, 2, 3, 4]. However, such studies highlight an increase in overall fall risk rather than specifically indicating an increased fall risk on stairs. Given that the UTTS requires balancing on a reduced base of support (i.e., forefoot region of the feet) and therefore relies on substantial contribution of the muscles around the ankle, that is, the functional output at the ankle joint, and given that ankle strength and function are related to fall risk [49, 50], functional mobility [30, 51] and stair negotiation [19, 20, 29]. It is possible that outcome measures from the UTTS test may provide a more sensitive means to identify those with an increased falling risk compared to the more traditional evaluation of the postural stability/sway during quiet standing. However, future work is needed to confirm this. Such future work should consider whether measures from the UTTS test provide a means to identify those with an increased risk of falling in general or are more specific to an increased risk of falling on stairs.

Although the current study demonstrates that balance‐related UTTS test scores differ between groups with expected differences in foot–ankle functioning, some limitations must be acknowledged. The first limitation is related to the lack of ankle/foot strength and passive range of motion measurements, foot sensation evaluation (proprioception) or visual assessments, which meant the study was unable to determine if the poorer UTTS performance in older adults with DPN was wholly due to reduced ankle strength, flexibility and functioning as opposed to being also partly due to sensory/visual impairments [52]. The second limitation was the control group being younger than the DPN group. Although the age difference between groups was in keeping with achieving the study's main aim, it should be acknowledged that postural control is influenced by ageing and the disease. Thus, the poorer test outcomes in the DPN group may be due to their increased age or due to them having diabetic peripheral neuropathy or a mixture of both. The young adult group also had a wide spread of ages (i.e., 43.8 ± 22.5 years old), which should be noted as a limitation because one or two from this group would have had an age closer to the mean age of the older adults with DPN group (70.8 ± 10.1 years old). Another limitation was that other than asking participants to wear comfortable flat shoes, footwear was not specifically controlled. It is widely known that older adults with DPN are at risk of foot ulceration [53] and as such specifically designed (diabetic) shoes or shoe inserts are recommended [54]. These shoes can alter the posture/angle of the foot and the foot's pressure distribution, which can potentially influence foot and ankle functioning [55, 56]. Future work is thus needed to determine if these types of shoes have any effect on UTTS performance. Finally, although the power of our statistical analysis was satisfactory, other studies analysing larger DPN samples would make the study more generalisable.

### 4.1 Conclusion

The UTTS test relies on substantial contribution of the muscles around the ankle, that is, the functional output at the ankle, which is a parameter known to be related to functional mobility, stair negotiation, and fall risk. The study demonstrated that older adults with DPN (i.e., persons with known impaired foot–ankle functioning) had significantly poorer UTTS test performance compared to young adults, which suggests the test may be useful for highlighting how deficits in ankle mobility and functioning impact balance. Future research should compare (a) older DPN patients with healthy older adults, and (b) young adults with older adults, to determine whether UTTS test can validly assess age‐related decline. In addition, case–control designs within the same age group are necessary to determine whether the UTTS test can distinguish disease‐specific balance deficits. Future studies are also needed to determine if impairments in test performance are associated with an increased risk of falling.

## Author Contributions

**John G. Buckley:** conceptualisation, formal analysis, methodology, project administration, visualisation, writing – original draft, writing – review and editing. **Asma Sabouni:** investigation, writing – review and editing. **Andre L.F. Rodacki:** conceptualisation, writing – review and editing. **Steve Brown:** conceptualisation, investigation, project administration, writing – review and editing. **Neil D. Reeves:** conceptualisation, writing – review and editing.

## Funding

The authors have nothing to report.

## Ethics Statement

Ethical approval was granted by the institutional bioethics committee (approval number, E.119) and the NHS Research Ethics Committee (18/NW/0274), and the tenets of the Declaration of Helsinki were observed.

## Consent

All participants gave their written informed consent to take part.

## Conflicts of Interest

The authors declare no conflicts of interest.

## Permission to Reproduce Material From Other Sources

The authors have nothing to report.

## Data Availability

The data that support the findings of this study are available from the corresponding author upon reasonable request.
